# Microwave Heating as an Innovative Road Maintenance Technology: Aging Effect on Binder and Feasibility Evaluation

**DOI:** 10.3390/ma15010316

**Published:** 2022-01-02

**Authors:** Aimin Sha, Baowen Lou, Diego Maria Barbieri, Inge Hoff

**Affiliations:** 1 School of Material Science and Engineering, Chang’an University, Nan Er Huan Road (Mid-Section), Xi’an 710064, China; ams@chd.edu.cn; 2 Department of Civil and Environmental Engineering, Norwegian University of Science and Technology, Høgskoleringen 7A, 7491 Trondheim, Norway; diego.barbieri@ntnu.no (D.M.B.); inge.hoff@ntnu.no (I.H.)

**Keywords:** microwave heating technique, asphalt aging, linear amplitude sweep, feasibility analysis

## Abstract

The microwave heating/healing technique is regarded as a green maintenance approach for asphalt pavements thanks to its promising environmental and economic benefits. However, the main concern about this technology is represented by the possible aging effect generated on bituminous binders. Currently, there is a significant lack of studies dealing with this topic. Based on these premises, the main purpose of this study is to appraise the feasibility of implementing microwave-based maintenance operations considering the associated aging effect. The assessment of fatigue life after cyclic microwave heating (MH) based on a linear amplitude sweep (LAS) test and the changes in the chemical groups detected through Fourier transform infrared (FTIR) spectroscopy document the aging phenomenon. The results indicate that the microwave aging degree on bituminous binder is nonlinear with MH cycles. The microwave radiation causes a distinct aging impact on binders during the first 10 cycles, then the values become constant. Furthermore, a feasibility analysis of MH technology is developed, encompassing four main multidisciplinary aspects: evaluation of microwave aging degree, working mechanism of MH equipment, safety assessment, and economic and ecological considerations. Despite the associated aging issue, the MH method is an efficient technology, considering its various advantages (i.e., rapidity of execution, uniform and non-pollutant treatment, and deep penetration). Meanwhile, the use of steel slag as a microwave absorber bolsters the sustainability of MH technology. This study provides a new perspective to evaluate the microwave heating technique in road engineering comprising the generated aging effect. Practice-oriented recommendations are also formulated regarding the safe implementation of MH technical operations.

## 1. Introduction

Asphalt materials are widely employed to construct surface layers of paved roads worldwide, thanks to the numerous advantages reported: it increases driver safety (high skid resistance and good road visibility), ensures a smooth, uniform surface, and provides a cost-effective, durable solution [1]. In general, the average lifespan of asphalt pavements can reach up to approximately 25 years with scheduled maintenance and rehabilitation operations. In recent years, the self-healing property of asphalt pavement has received great attention as a means to repair the fatigue cracks and restore the stiffness and strength during the rest period of pavement [2]. Based on the increasing traffic volume and demand for long-lasting roads, the introduction of external heating interventions would be beneficial to accelerate this healing process.

Compared with other heating methods (i.e., hot air, flame, infrared, electric coils, and hydronics), the microwave heating (MH) technique is considered as an innovative maintenance solution due to its rapid, uniform, non-polluting, and deep penetrating heating characteristics [3,4,5]. Microwaves are a form of electromagnetic waves in the frequency range of 300 MHz to 300 GHz [6], which generate a volumetric heating phenomenon [7]. When it comes to their field application, the high-frequency, reciprocating motion of internal dipole molecules promotes the generation of internal friction heat under the microwave radiation [8,9]; meanwhile, the flow and diffusion of asphalt binder contributes to crack repair at high temperatures. However, the microwave-absorbing ability of conventional asphalt concrete is quite limited due to its low susceptibility; thus, microwave absorbers (i.e., steel slag [10], metallic fibers [11], and carbon nanomaterials [12]) can be added into mixtures to enhance the conversion efficiency of electromagnetic energy into thermal energy. The microwave absorber considered in this study is steel slag. Its use in road pavement infrastructures can not only improve the road performance [13,14], but also represents an environmentally friendly practice as it prevents or reduces the consumption of natural resources [15]. In order to satisfy the volume stability of steel slag, some pretreatment methods, such as cooking and autoclave [16], should be adopted to reduce the content of free CaO to less than 3% [17]. Previous research has indicated the physical properties of steel slag can fulfil the road performance requirements [18,19], while more technical and environmental studies need to be carried out before employing steel slag in actual asphalt pavements. Furthermore, the MH technique can also be successfully applied, considering recycling reclaimed materials, pothole patching, and deicing [20,21,22].

Nevertheless, the main concern of this quick heating method is related to the aging issue: on the one hand, uneven heating of asphalt pavement may cause charring of surface asphalt binder [23]; on the other hand, cyclic MH treatment could led to the aging of asphalt binder. With the development of technology and material innovation, the non-uniform heating issue can be properly solved, while the microwave aging impact is unavoidable. Some research preliminarily indicated that the MH may trigger aging of the asphalt binder [24,25,26], but the current studies specifically delving into the topic are still limited. Based on this premise, this research evaluates the aging generated by MH technology by investigating the fatigue performance of asphalt binder used for road surface layer.

Asphalt binders are viscoelastic materials used to produce asphalt mixtures, which can resist distresses, such as rutting and cracking, and somehow dictate the performance and the serviceable life of pavement [27]. The dynamic shear rheometer (DSR) is able to quantify both elastic and viscous properties, and this device is widely used to characterize the rheological properties of asphalt binder, for instance, its complex shear modulus (G*) and phase angle (δ). In recent years, the time sweep (TS) test and linear amplitude sweep (LAS) test have been developed to accurately determine the fatigue resistance of asphalt binder considering the damage accumulation with repeated loading. The TS test is operated according to several repeated load cycles under a constant amplitude, frequency, and temperature [27]. The failure point in this strain-controlled test is reached when the complex modulus/dissipated energy drops by 50% of the initial value [28], and the number of cycles to failure is recorded as fatigue life. Although this type of investigation presents a good correlation with the fatigue life of mixtures and is reliable for binder fatigue evaluation, the main drawback of this method is that it is a time consuming test [29,30]. Therefore, the LAS test is adopted to accelerate damage accumulation and replace the TS approach, and the testing period can be shortened to approximately 5 min. Therefore, this study sheds light on the aging degree of microwave heated asphalt binder and associated changes in fatigue life by performing LAS tests. The four main classes of compounds of asphalt components include naphthene aromatics, polar aromatics, saturated hydrocarbons, and asphaltenes, and they are greatly affected by the aging extent of asphalt. Thus, since both the microwave energy and aging are phenomena related to the molecular level [31], this research employs Fourier transform infrared (FTIR) spectroscopy to detect the chemical functionalities of aged binder by measuring the absorbance intensity of carbonyl groups.

**Figure 1:**
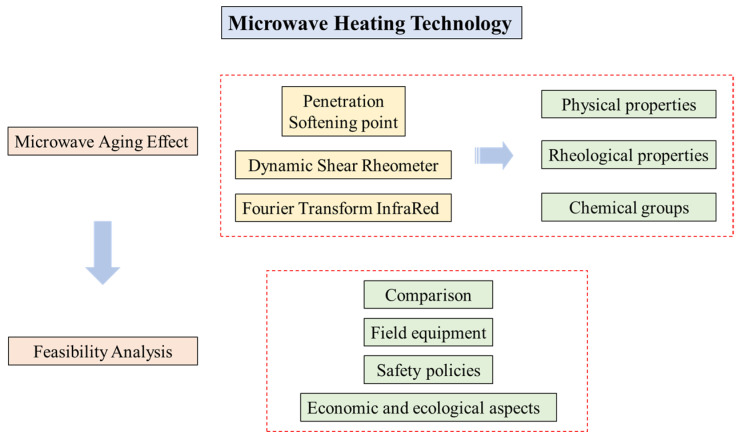
Flowchart of the research approach.

In summary, the main objective of this study is to appraise the feasibility of applying the MH technology in road construction. To fulfill this objective (as shown in Figure 1), the asphalt binders are firstly extracted from mixtures aged with a series of MH cycles. LAS and FTIR tests are then employed to evaluate the aging extent of the bituminous binder, analyzing the rheological properties and the chemical groups, respectively. In addition, this study demonstrates the development of the LAS test method and documents the feasibility of MH applications in the field. Overall, this research provides a comprehensive analysis of MH technology considering the microwave aging issue.

## 2. Background

The fatigue performance of asphalt concrete mainly depends on the binder/mastic phase, and its fatigue resistance can be evaluated via accelerated damage progress based on the continuum damage principle [32]. LAS tests are performed in this study to estimate the fatigue resistance of asphalt binder according to AASHTO TP 101 [33]. The procedure is firstly performed in shear using a frequency sweep to determine the rheological properties, and then a series of oscillatory load cycles at systematically increasing amplitudes at a constant frequency are applied to accelerate damage in the specimen. The damage accumulation for the LAS test can be used to determine the fatigue resistance from the results of both the frequency sweep and amplitude sweep, adopting the following fatigue damage model with coefficients *A* and *B*:

(1) $$N_{f}=A\cdot {\left(\gamma_{max}\right)}^{-B}$$

where *N_f_* is the fatigue life and $\gamma_{max}$ (%) is the maximum expected binder strain for a given pavement structure.

In order to calculate parameters *A* and *B* of equation (1), we employ the concept of viscoelastic continuum damage (VECD), which was firstly proposed in 1984 for asphalt mixtures [34] to predict damage evolution, which was primarily characterized through the deviations from linear viscoelastic behavior and stiffness reduction. Subsequently, a large number of research efforts have proven the accuracy of the parameters derived from this formulation to predict the damage evolutions in asphalt materials, irrespective of the testing temperature or loading mode [35,36,37,38,39]. In this regard, this approach has become one of the most widely used mechanistic models.

Based on Schapery’s theoretical work, a relationship between material integrity (*C*) and damage intensity (*D*) is established and it describes how the damage accumulation affects the loss of the structural integrity during the cyclic loading [40]. According to the damage characteristic curve (DCC) for a given pavement structural condition or traffic loading, the damage accumulation rate can evaluate the fatigue performance of asphalt binders. Meanwhile, the relationship between *C* and *D* is independent of the test conditions, making it widely used in investigating the fatigue life of asphalt materials [41]. The damage evolution is represented in Equation (2):

(2) $$\frac{dD}{dt}={\left(-\frac{\partial W}{\partial D}\right)}^{\alpha }$$

where *W* denotes the performed work, *t* is time, and α equals 1 + 1/*m*. *α* is a material-dependent parameter describing the damage growth and it can be calculated using the slope of the storage modulus versus the angular frequency in log-space (log *G*′(*ω*) = *m*(log *ω*) + *b*) [42].

To represent *W* in Equation (2), Kim et al. took the energy dissipated during each load cycle into consideration [43]. The damage calculation under strain-controlled cyclic shear loading is shown in Equation (3) and the damage accumulation as a function of time can be described according to Equation (4):

(3) $$W=\pi \cdot I_{D}\cdot \gamma_{0}^{2}\cdot \left|G^{*}\right|\cdot sin\delta$$

(4) $$D\left(t\right)≅\overset{N}{\underset{i=1}{\sum }}{\left[\pi I_{D}\gamma_{0}^{2}\left(\left|G^{*}\right|sin\delta_{i-1}-\left|G^{*}\right|sin\delta_{i}\right)\right]}^{\frac{\alpha }{1+\alpha }}{\left(t_{i}-t_{i-1}\right)}^{\frac{1}{1+\alpha }}$$

where $I_{D}$ (MPa) is the initial undamaged dynamic shear modulus divided by a modulus of 1 MPa, $\gamma_{0}$ (%) refers to the applied strain for a given data point, $\left|G^{*}\right|$ (MPa) corresponds to the complex shear modulus, and δ (°) is the phase angle.

Fatigue tests are performed at different applied strain amplitudes with a 1.0 % interval, and the incremental value of D(t) at each subsequent point is added to the value of D(t) from the previous point until a total of 30% applied strain is reached. The value of $\left|G^{*}\right|sin\delta_{i}$ is plotted against the corresponding value of $D\left(t_{i}\right)$ based on a power law [34,44] as detailed in Equation (5):

(5) $$\left|G^{*}\right|sin\delta =C_{0}-C_{1}{\left(D\right)}^{C_{2}}$$

where $C_{0}$ is the average value of $\left|G^{*}\right|\cdot sin\delta$ from the 0.1% strain interval, and *C_1_* and *C_2_* are regression coefficients.

Afterwards, Equation (5) is substituted into Equation (3) and the derivative is taken with respect to *D* as shown in Equation (6). This expression is then combined with Equation (2) to determine the number of cycles to failure given a value of the damage parameter *D* at failure as shown in Equation (7):

(6) $$\frac{dW}{dD}=-\pi I_{D}C_{1}C_{2}{\left(D\right)}^{C_{2}-1}{\left(\gamma_{max}\right)}^{2}$$

(7) $$N_{f}=\frac{f{\left(D_{f}\right)}^{k}}{k{\left(\pi I_{D}C_{1}C_{2}\right)}^{\alpha }}{\left(\gamma_{max}\right)}^{-2\alpha }$$

where *k* = 1 + (1 − *C*_2_)*α*, *f* (Hz) is the loading frequency and *D_f_* refers to the damage accumulation at failure.

Grouping the following parameters in Equation (7), the fatigue equation in Equation (1) can be expressed referring to:

(8) $$A=\frac{f{\left(D_{f}\right)}^{k}}{k{\left(\pi I_{D}C_{1}C_{2}\right)}^{\alpha }}$$

(9) B=2α

In terms of VECD model coefficients, it can be directly observed from Equation (1) that the parameter *A* is equal to the fatigue life of the binder at 1% strain level.

## 3. Materials and Methods

### 3.1. Raw Materials

**Figure 2:**
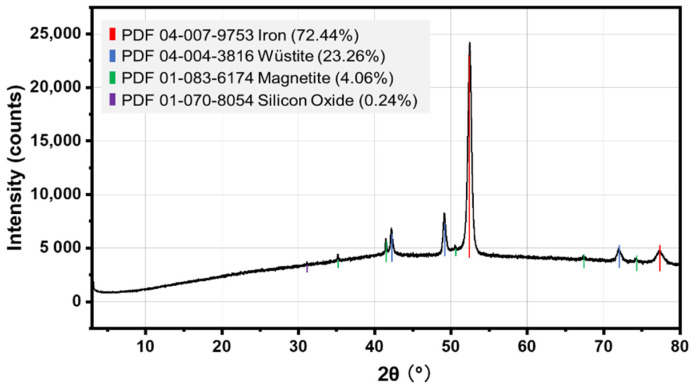
X-ray diffraction pattern of the steel slag.

A neat base asphalt binder with a penetration of 82 dmm, a softening point of 46.3 °C, dynamic viscosity of 188 Pa·s at 60 °C, and a density of 1.01 g/cm^3^ at 25 °C is used in this study. The aggregates include crushed rocks and steel slag, and are provided by Franzefoss Pukkverk (Heimdal, Norway) and Skanska Stålfabrikk (Melhus, Norway), respectively. The rock aggregates are mainly composed of fine-grained gabbro/metagabbro and are widely used for road construction in the central part of Norway [45,46]. As a typical magnetic material, steel slag is adopted as a microwave absorber in the asphalt mixtures to generate heat. According to the X-ray diffraction pattern of the steel slag illustrated in Figure 2, the existence of metallic composites proves that the material can help to increase the microwave heating capacity of the mixtures.

As a traditional road surfacing material commonly used in Norwegian conditions, standard AC-11 dense asphalt mixtures are fabricated [47]. The prepared samples are then treated under the microwave radiation until the average temperature reaches 90 °C, which is considered a proper heating temperature to guarantee the asphalt binder a flow state without overheating. A domestic microwave oven with output power of 700 W and working frequency of 2.45 GHz is adopted. In this study, 3, 5, 10, 15, 20, and 25 MH cycles (namely ^#^3, ^#^5, ^#^10, ^#^15, ^#^20, and ^#^25) are performed to evaluate the aging effect.

### 3.2. Extraction of Asphalt Binder

**Figure 3:**
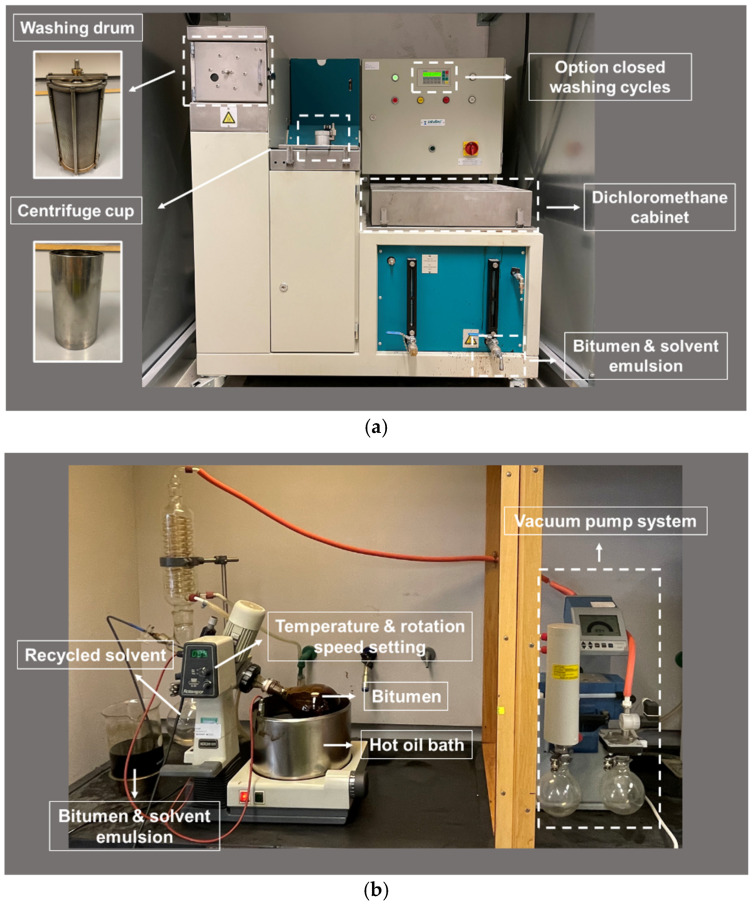
Extraction acquisition process of aged asphalt binder. (**a**) Extraction of asphalt binder and dichloromethane solvent from the mixtures; (**b**) Rotary evaporation.

The aged asphalt binder is extracted from the mixtures through an extraction machine (Asphalt Analyzer YOU, InfraTest, Brackenheim, Germany), together with dichloromethane solvent. The crumbled asphalt samples are placed into a washing drum and undergo a series of eight washing cycles, an amount selected to make sure that the asphalt binder can be totally extracted from the mixtures (Figure 3a). Subsequently, the acquired bitumen and dichloromethane mixtures are separated by employing a rotary evaporator (Buchi, Flawil, Switzerland), thanks to their different evaporation temperatures (Figure 3b). Finally, the pure aged asphalt binder can be collected and further analyzed.

### 3.3. LAS Test

The LAS test is used to determine the fatigue damage of asphalt binder by means of cyclic loading employing systematic, linearly increasing load amplitudes. The LAS tests are operated with a dynamic shear rheometer machine (DSR, Anton Paar, Graz, Austria) using the 8 mm parallel plate geometry with a 2 mm gap setting. The LAS test involves two steps: a frequency sweep is firstly performed to evaluate the rheological properties of asphalt binder, then an amplitude sweep is employed to measure the damage characteristics. As mentioned in Section 2, the damage analysis parameter “*α*” can be deduced during the frequency sweep, which possesses an applied load of 0.1% strain over a range of frequencies from 0.2 to 30 Hz. Subsequently, the amplitude sweep is run in strain-control mode at a frequency of 10 Hz using oscillatory shear, and the amplitude is linearly increased from 0.1% to 30% in 5 min. The failure definition in the LAS test corresponds to a 35% reduction in the initial modulus.

### 3.4. Infrared Spectrum Test

The microstructural characteristics are captured by an FTIR spectrometer (Nicolet iS50, ThermoFisher, Waltham, MA, USA) within the scan range of 4000 cm^−1^–400 cm^−1^. In order to assess the microwave aging effect on asphalt binders, the two commonly used aging parameters carbonyl index and sulfoxide index are adopted, as shown in Equations (10) and (11) [48,49].

(10) $$I_{C=O}=\frac{\mathrm{Characteristic}\;\mathrm{peak}\;\mathrm{area}\;\mathrm{around}\;1700{\;\mathrm{cm}}^{-1}\;\mathrm{carbonyl}\;\mathrm{group}}{\mathrm{Sum}\;\mathrm{peak}\;\mathrm{area}\;\mathrm{between}\;2000{\;\mathrm{cm}}^{-1}\;\mathrm{and}\;600{\;\mathrm{cm}}^{-1}\;}$$

(11) $$I_{S=O}=\frac{\mathrm{Characteristic}\;\mathrm{peak}\;\mathrm{area}\;\mathrm{around}\;1030{\;\mathrm{cm}}^{-1}\;\mathrm{sulfoxide}\;\mathrm{group}}{\mathrm{Sum}\;\mathrm{peak}\;\mathrm{area}\;\mathrm{between}\;2000{\;\mathrm{cm}}^{-1}\;\mathrm{and}\;600{\;\mathrm{cm}}^{-1}\;}$$

## 4. Results and Discussions

### 4.1. Fatigue Performance of Microwave Aged Asphalt

**Figure 4:**
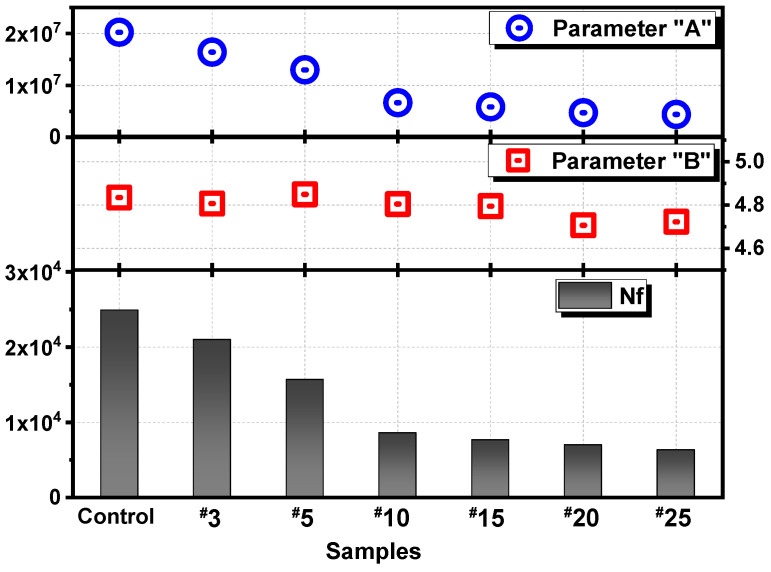
Parameters in LAS tests and fatigue life (*N_f_*) at 4% strain level.

The parameters *A* and *B* can be obtained from the accelerated LAS test to evaluate the fatigue resistance. The value of the storage modulus is reduced during the process of the amplitude sweep, indicating the decreasing ability of the asphalt binder to maintain its integrity during loading cycles and the accumulated damage process, and this trend can be represented by parameter *A* [50]. Meanwhile, parameter *B* is related to the sensitivity of the asphalt binder to strain level change, and a higher value of this parameter indicates that the fatigue life decreases at a greater rate when strain level amplitude is augmented. In summary, a better fatigue resistance ability of asphalt binder is associated with a higher *A* value and lower *B* value [51]. As Figure 4 shows, parameter *A* clearly decreases with the increase in MH cycles. *N_f_* follows a similar trend, which demonstrates that the fatigue parameter *A* plays a primary role in determining the fatigue life of asphalt binder.

In addition, *N_f_* is calculated at 4% strain level as previous studies pointed out that the fatigue failure does not occur at very low strain levels [52]. Considering Figure 4, the *N_f_* value of the control unaged asphalt sample is approximately 1.2, 1.6, and 2.9 times larger than the aged samples undergoing microwave action three (^#^3), five (^#^5), and ten (^#^10) times. The cyclic MH process causes a visible reduction in the *N_f_* value; however, when the heating treatment exceeds 10 cycles, the difference exerted by different amounts of MH cycles no longer becomes obvious. This nonlinear effect of MH on the fatigue resistance of asphalt binder is, to some extent, unexpected, as the more MH cycles performed, the smaller the impact on the rheological properties. This phenomenon is highly relevant when it comes to estimating the feasibility of MH applications in the field. To better understand the MH influence on asphalt binder, the changes in chemical groups should be detected through the FTIR method.

### 4.2. Infrared Spectroscopy of Microwave Aged Asphalt

**Figure 5:**
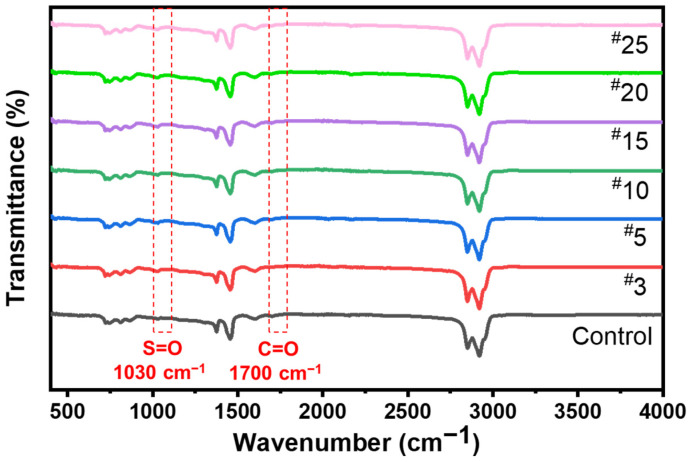
FTIR results of asphalt with different MH cycles.

Figure 5 shows the FTIR spectra of microwave-treated asphalt samples; overall, the spectra curves are similar. The main oxygen-containing functional groups, namely sulfoxide (S=O) and carbonyl (C=O), appearing at 1700 cm^−1^ and 1030 cm^−1^, respectively, are adopted as the main indicators of asphalt aging. The aging indexes I_C=O_ and I_S=O_ have a similar changing trend as presented in Table 1: the aging index of the sample treated with 10 microwave cycles (#10) is approximately 1.2 times higher than the index of the control specimen. The increase in I_S=O_ index does not follow a precise trend, while the I_C=O_ of ^#^15, ^#^20, and ^#^25 is about 1.29, 1.40, and 1.50 times larger than the control sample. This minor difference between the above two aging indexes after 10 MH cycles may be attributed to the fact that the microwave energy causes chemical changes in asphalt at the molecular level [22], which would cause a deviation in the determination of oxidative aging parameters. In summary, the FTIR results are consistent with the characteristics of MH on the asphalt aging observed from the LAS tests.

### 4.3. Comprehensive Feasibility Analysis

The potential use of microwave energy to heat and maintain asphalt road pavements has attracted increasing attention recently. Even if much work has been done regarding the microwave absorbers selection, as well as their efficiency and mechanisms, there is a noticeable paucity of information available in the literature about the feasibility of applying MH technologies in real scenarios. Therefore, a practice-oriented discussion regarding the feasibility of MH implementation is presented in the following four subsections.

#### 4.3.1. Practicability of MH Technology Considering Aging

**Figure 6:**
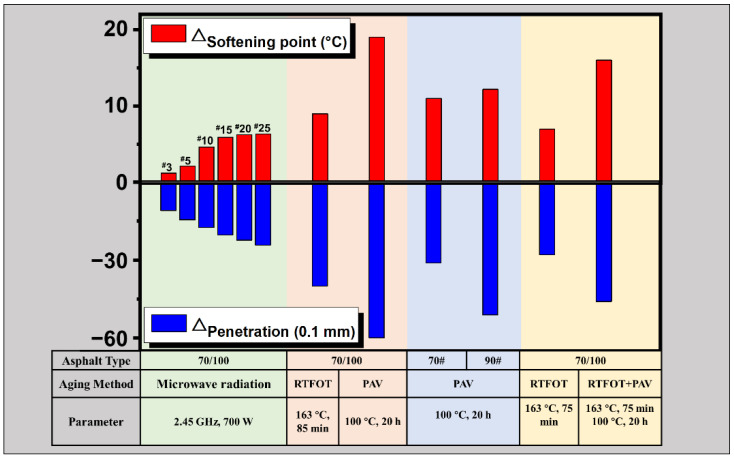
Comparison of asphalt aging effect of the MH method with other aging data acquired from [55,56,57], respectively.

In Section 4.1 and Section 4.2, laboratory study is conducted to estimate the microwave aging effect on asphalt binders by characterizing the changes in the rheological properties and molecular structures. Considering the fatigue lives derived from the LAS tests, the microwave radiation does cause aging on asphalt binders, especially during the initial applied MH cycles. Under the conditions of this study, an obvious deterioration of fatigue lives can be observed within 10 MH cycles, while minor changes can be detected during further MH cycles, and the aging indexes acquired from FTIR agree well with this trend. To intuitively analyze the extent of the aging degree caused by microwave radiation, Figure 6 compares the penetration and softening point parameters obtained in this study with other findings reported in the literature testing similar asphalt type with different aging methods according to NS-EN 1426 and NS-EN 1427 [53,54]. Considering the results attained in this study after cyclic MH treatment, the values follow the similar changing trend as *N_f_* indicated in LAS tests. The changes in physical properties are relatively apparent during the first few cycles and then level off after 10 cycles. When comparing these outcomes with the laboratory aging data collected from other tests [55,56,57], the deterioration reported by MH technology is not considerably significant. Therefore, bearing in mind the various benefits of MH technology (rapidity of execution, uniform and non-polluting treatment, deep penetration), the aging impact on asphalt binders is within an acceptable range. Furthermore, other interventions, such as adding encapsulated rejuvenators, adjusting microwave heating parameters, and selecting the proper microwave maintenance frequency, could hinder aging, to some extent.

#### 4.3.2. Practicability of MH Technology Considering Field Equipment

**Figure 7:**
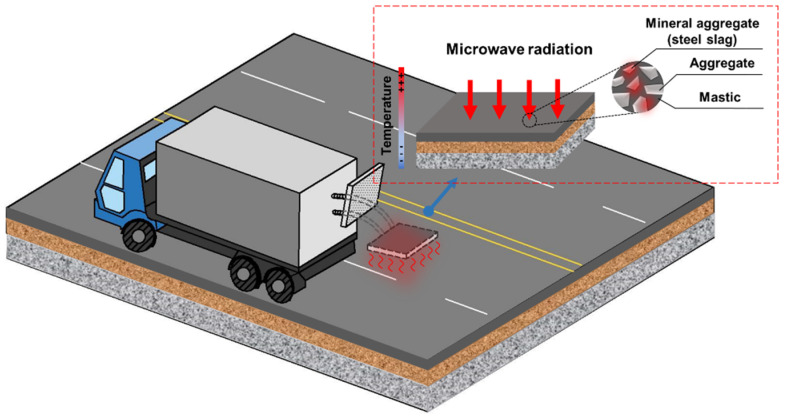
Diagram of MH equipment.

The early practical use of MH energy in pavement engineering was envisaged to rapidly patch Portland cement concrete with polymer modifiers [58]. The microwave device comprised eight radiating units (2.5 kW for each unit) and the frequency was equal to 2.45 GHz, which could radiate an area of 0.73 m × 1.1 m. Afterwards, a large 100 kW and 915 MHz microwave asphalt pavement heater was built in California to patch potholes and repair longitudinal cracks, and the applicators were enclosed by a curtain made of closed-spaced metal chains. In Texas, a microwave recycling system was developed to heat reclaimed asphalt pavement: the target pavement areas were firstly preheated in a fluidized bed warm-air oven using a conventional burner, then the materials were conveyed into a microwave radiation tunnel to reach the desired working temperature. The 200 kW power of the microwave tunnel could guarantee a maximum production rate of 200 tons per hour, which led to a promising 100% ratio of recycled asphalt concrete mix in Austin, Texas in the summer of 1986 [59]. In recent years, asphalt road microwave maintenance vehicles have also been developed in China (WB series) and used to repair potholes, fatigue and block cracking, grooves, etc. in asphalt highways, local roads, and even airport runways [60]. Overall, the MH technique can be applied for different purposes related to asphalt pavements, and the fundamental MH field equipment is a mobile, truck-mounted microwave power generator. A general schematic diagram is depicted in Figure 7. The MH panel at the rear of the vehicle slows down on the pavement area to be treated and the microwave units heat the pavement. Meanwhile, the MH vehicle can be followed by a compactor roller to heal cracks, a recycler machine to collect reclaimed asphalt, a deicing machine to mince ice crust, etc.

#### 4.3.3. Practicability of MH Technology Considering Safety Policies

Introducing the innovative MH technique to perform maintenance operations of road pavement constructions requires special attention to thoroughly address unforeseen challenges and multifaceted safety issues. Although, currently, there are no specific standards that regulate possible leakages originating from microwave heating machines for paving purposes, this issue can be discussed here considering the code framework actually in force regarding domestic microwave ovens. Several relevant institutions, i.e., the International Electrotechnical Commission (IEC), the International Committee on Electromagnetic Safety (ICES), and the European Committee for Electrotechnical Standardization (CENELEC), define a product emission limit of 50 watts per square meter (W/m^2^) at any point 5 cm away from the external surfaces of the oven [61]. In general, emissions from the microwave units are substantially below this international limit and the exposure decreases dramatically with distance. When it comes to the field application of MH, many safety precautions should be adopted: the microwave applicators should be surrounded by a metallic enclosure with a lock system which is part of the on-off switching mechanism, and a remote controlled switching arrangement could further increase the safety level [58]; the applicators should be enclosed by a curtain made of closely spaced, short vertical metal chains in Microdry systems [20]; and Hertzian waves radiation shield technology should be adopted to keep the radiation to low levels to guarantee high security [60]. Therefore, thanks to the introduction of the metal shields, remote control, and monitor systems, MH can be considered as a safe technique, though it is still suggested to (1) regularly clean the MH panel, (2) guarantee the MH unit is in good condition before use, and (3) avoid standing directly against the MH panel when it is operational.

#### 4.3.4. Practicability of MH Technology Considering Economic and Ecological Aspects

**Figure 8:**
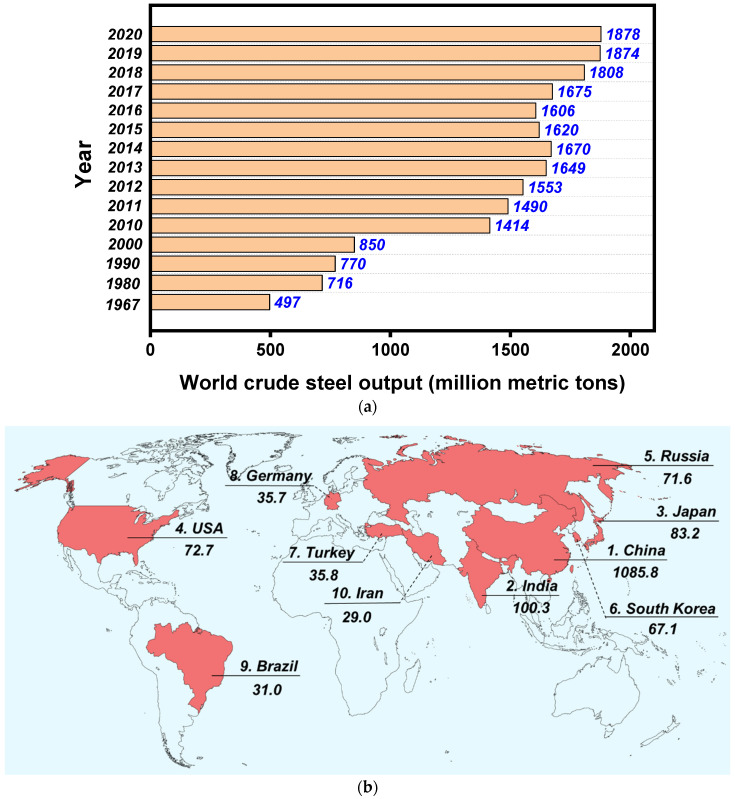
(**a**) Production of world crude steel and (**b**) its distribution in 2020 (million metric tons).

The economic and ecological values of MH are attributed to its uniform, deep, and fast heating characteristics, which can effectively reduce construction time, leading to savings in labor cost and reduced traffic disruptions [62]. For example, based on an open life cycle assessment framework, Ali-Mansoori et al. indicated that there was a remarkable reduction in greenhouse gas emission and energy usage (approximately 22 GJ) in microwave heating pavements compared with conventional pavements [63]. Compared with other hot-in-place recycling methods, 40% to 50% energy can be saved under the first generation of microwave pavement heaters in a field test [64]. Therefore, MH can be regarded as an innovative and environmentally friendly technology for road maintenance operations, which can not only reduce the cost and extend the service life of the pavement, but can also decrease the polluting emissions. Beyond being a typical microwave absorber, steel slag is a byproduct of metallurgical industries. According to the World Steel Association report [65], the steel slag industry is at the heart of the global economy of both developed and developing countries and the amount of steel production is steadily increasing, as reported in Figure 8; consequently, a large quantity of its byproducts (e.g., steel slag) needs to be properly managed. Therefore, the incorporation of steel slag in asphalt pavement can reduce the construction cost of MH pavement [21], as well as reduce the associated environmental burdens and save natural resources.

## 5. Conclusions

This research has appraised the MH technique according to two main perspectives: evaluation of the aging impact on steel slag asphalt mixtures, and practice-oriented feasibility assessment of MH application.

The study has performed laboratory tests, including the evaluation of physical properties (penetration point, softening point) and rheological properties (fatigue life), as well as identification of chemical groups (infrared spectra) to investigate the changes in microwave heated samples. The findings document that the MH technique causes aging and its impact is nonlinear with the amount of MH cycles. The fatigue lives, calculated with an LAS test based on the VECD model, are characterized by a relatively distinct deterioration during the first 10 cycles. The aging indexes assessed with FTIR tests and physical tests also document the aging effect and agree well with the trend of the calculated fatigue lives.

Furthermore, a comprehensive feasibility analysis has been proposed, revolving around four main aspects: evaluation of the microwave aging degree, description of MH equipment for field implementation, development of safety policies, and the discussion of economic and ecological aspects. Despite the fact that asphalt aging is the main concern of MH technology, its field implementation is still feasible and cost-effective based on the following considerations:

1. The microwave irradiation causes aging of asphalt binder. However, the aging effect is not significant and it tends to level off after several cycles.
2. Other interventions, such as adding encapsulated rejuvenators [66,67], adjusting microwave heating parameters [68], and selecting appropriate microwave maintenance frequency [69], can hinder aging, to some extent.
3. As every newly developed technology has its own pros and cons, future research should focus on maximizing the advantages, while unraveling the drawbacks and preventing detrimental effects to human health and the total environment.

Moreover, policies regarding safety issues and the use of well-designed MH equipment should be borne in mind to prevent any harm to operators. Finally, a wise selection of the microwave absorbers inside the road pavement, i.e., steel slag, can lead to additional economic and ecological benefits.

In summary, this study provides a useful reference documenting the aging effect and the practicality of microwave technology for the maintenance of asphalt roads. Before implementing the technology in the field, it is recommended that laboratory and large-scale tests are performed to determine all the relevant technical parameters. In the future, more practice-oriented research should focus on the laboratory standardization of microwave heating/healing efficiency, as well as full-scale implementation.

## Figures and Tables

**Table 1:** I_S=O_ and I_C=O_ changes of asphalt after microwave aging.

Index	Control	^#^3	^#^5	^#^10	^#^15	^#^20	^#^25
I_S=O_	0.0143	0.0163	0.0169	0.0173	0.0177	0.0189	0.0194
I_C=O_	0.0065	0.0069	0.0071	0.0076	0.0083	0.0090	0.0097

## Data Availability

The data can be found by contacting the corresponding author.

## References

[1] González A., Norambuena-Contreras J., Storey L., Schlangen E. (2018). Effect of RAP and fibers addition on asphalt mixtures with self-healing properties gained by microwave radiation heating. Constr. Build. Mater..

[2] Pasupunuri S., Tiwari D., Jain S., Kumar P. Self-Healing Pavements: A Revolution in Pavement Materials. Proceedings of the IRF World Road Meeting.

[3] Xu X., Gu H., Dong Q., Li J., Jiao S., Ren J. (2018). Quick heating method of asphalt pavement in hot in-place recycling. Constr. Build. Mater..

[4] Ho I.H., Dickson M. (2017). Numerical modeling of heat production using geothermal energy for a snow-melting system. Geomech. Energy Environ..

[5] Sun T. (2013). Thermoelectric coupling model for asphalt mixtures based on microwave heating. Int. J. Appl. Electromagn. Mech..

[6] Gallego J., del Val M.A., Contreras V., Paez A. (2013). Heating asphalt mixtures with microwaves to promote self-healing. Constr. Build. Mater..

[7] Gulisano F., Gallego J. (2021). Microwave heating of asphalt paving materials: Principles, current status and next steps. Constr. Build. Mater..

[8] Li C., Wu S., Chen Z., Tao G., Xiao Y. (2018). Enhanced heat release and self-healing properties of steel slag filler based asphalt materials under microwave irradiation. Constr. Build. Mater..

[9] Lou B., Sha A., Barbieri D.M., Liu Z., Zhang F., Jiang W., Hoff I. (2021). Characterization and microwave healing properties of different asphalt mixtures suffered freeze-thaw damage. J. Clean. Prod..

[10] Lou B., Sha A., Li Y., Wang W., Liu Z., Jiang W., Cui X. (2020). Effect of metallic-waste aggregates on microwave self-healing performances of asphalt mixtures. Constr. Build. Mater..

[11] Sun Y., Wu S., Liu Q., Zeng W., Chen Z., Ye Q., Pan P. (2017). Self-healing performance of asphalt mixtures through heating fibers or aggregate. Constr. Build. Mater..

[12] Wang Y., Liu Z., Hao P. (2019). Investigation on mechanical and microwave heating characteristics of asphalt mastic using activated carbon powder as electro-magnetic absorbing materials. Constr. Build. Mater..

[13] Wen H.F., Wu S.H., Bhusal S. (2016). Performance evaluation of asphalt mixes containing steel slag aggregate as a measure to resist studded tire wear. J. Mater. Civ. Eng..

[14] Xie J., Chen J.Y., Wu S.P., Lin J.T., Wei W. (2013). Performance characteristics of asphalt mixture with basic oxygen furnace slag. Constr. Build. Mater..

[15] Ferreira V.J., Vilaplana A.S.-D.-G., García-Armingol T., Aranda-Usón A., Lausín-González C., López-Sabirón A.M., Ferreira G. (2016). Evaluation of the steel slag incorporation as coarse aggregate for road construction: Technical requirements and environmental impact assessment. J. Clean. Prod..

[16] Wei L.H., Qi X.J., Zhu X., Wang H., Hu B., Xu Y., Li Y., Li X. (2017). Influence of pretreatment on the free CaO in Steel Slag. Materials Science Forum.

[17] (2010). Steel Slag for Road.

[18] Lou B., Liu Z., Sha A., Jia M., Li Y. (2020). Microwave absorption ability of steel slag and road performance of asphalt mixtures incorporating steel slag. Materials.

[19] Wan J., Wu S., Xiao Y., Fang M., Song W., Pan P., Zhang D. (2019). Enhanced ice and snow melting efficiency of steel slag based ultra-thin friction courses with steel fiber. J. Clean. Prod..

[20] Al-Ohaly A.A. (1987). Laboratory Evaluation of Microwave Heated Asphalt Pavement Materials.

[21] Gao J., Sha A., Wang Z., Tong Z., Liu Z. (2017). Utilization of steel slag as aggregate in asphalt mixtures for microwave deicing. J. Clean. Prod..

[22] Lou B., Sha A., Barbieri D.M., Liu Z., Zhang F., Jiang W. (2021). Improved microwave heating uniformity and self-healing properties of steel slag asphalt containing ferrite filler. Mater. Struct. Mater. Constr..

[23] Li X., Ma D., Yang S. (2016). Heating method for in-place heat recycling of asphalt pavement. J. Cent. South Univ..

[24] Franesqui M.A., Yepes J., García-González C. (2017). Top-down cracking self-healing of asphalt pavements with steel filler from industrial waste applying microwaves. Constr. Build. Mater..

[25] Norambuena-Contreras J., Gonzalez A., Concha J.L., Gonzalez-Torre I., Schlangen E. (2018). Effect of metallic waste addition on the electrical, thermophysical and microwave crack-healing properties of asphalt mixtures. Constr. Build. Mater..

[26] Zhu X., Cai Y., Zhong S., Zhu J., Zhao H. (2017). Self-healing efficiency of ferrite-filled asphalt mixture after microwave irradiation. Constr. Build. Mater..

[27] Hajj R., Bhasin A. (2017). The search for a measure of fatigue cracking in asphalt binders—A review of different approaches. Int. J. Pavement Eng..

[28] Shen S., Airey G.D., Carpenter S.H., Huang H. (2006). A dissipated energy approach to fatigue evaluation. Road Mater. Pavement Des..

[29] Martono W., Bahia H.U., D’angelo J. (2007). Effect of testing geometry on measuring fatigue of asphalt binders and mastics. J. Mater. Civ. Eng..

[30] Bonnetti K.S., Nam K., Bahia H.U. (2002). Measuring and defining fatigue behavior of asphalt binders. Transp. Res. Rec..

[31] Bishara S.W., McReynolds R. (1996). Laboratory aging and annealing of asphalt binders by microwave radiation. Transp. Res. Rec..

[32] Johnson C.M. (2010). Estimating Asphalt Binder Fatigue Resistance Using an Accelerated Test Method. Ph.D. Thesis.

[33] (2014). Standard Method of Test for Estimating Damage Tolerance of Asphalt Binders Using the Linear Amplitude Sweep.

[34] Schapery R.A. (1984). Correspondence principles and a generalized J integral for large deformation and fracture analysis of viscoelastic media. Int. J. Fract..

[35] Daniel J.S., Kim Y.R. (2002). Development of a simplified fatigue test and analysis procedure using a viscoelastic, continuum damage model (with discussion). J. Assoc. Asph. Paving Technol..

[36] Kutay M.E., Gibson N.H., Youtcheff J. (2008). Conventional and viscoelastic continuum damage (VECD)-based fatigue analysis of polymer modified asphalt pavements (with discussion). J. Assoc. Asph. Paving Technol..

[37] Lee H.-J., Kim Y.R. (1998). Viscoelastic continuum damage model of asphalt concrete with healing. J. Eng. Mech..

[38] Norouzi A., Richard Kim Y. (2017). Mechanistic evaluation of fatigue cracking in asphalt pavements. Int. J. Pavement Eng..

[39] Sabouri M., Kim Y.R. (2014). Development of a failure criterion for asphalt mixtures under different modes of fatigue loading. Transp. Res. Rec..

[40] Zhang H., Shen K., Xu G., Tong J., Wang R., Cai D., Chen X. (2020). Fatigue resistance of aged asphalt binders: An investigation of different analytical methods in linear amplitude sweep test. Constr. Build. Mater..

[41] Cao W., Wang C. (2018). A new comprehensive analysis framework for fatigue characterization of asphalt binder using the Linear Amplitude Sweep test. Constr. Build. Mater..

[42] Schapery R. (1990). A theory of mechanical behavior of elastic media with growing damage and other changes in structure. J. Mech. Phys. Solids.

[43] Kim Y., Lee H., Little D., Kim Y.R., Gibson N., King G., Pellinen T., Fee F. (2006). A simple testing method to evaluate fatigue fracture and damage performance of asphalt mixtures. Proceedings of the 2006 Journal of the Association of Asphalt Paving Technologists: From the Proceedings of the Technical Sessions.

[44] Schapery R.A. (1975). A theory of crack initiation and growth in viscoelastic media II. Approximate methods of analysis. Int. J. Fract..

[45] Barbieri D.M., Hoff I., Mørk M.B.E. (2020). Organosilane and lignosulfonate as innovative stabilization techniques for crushed rocks used in road unbound layers. Transp. Geotech..

[46] Barbieri D., Hoff I., Mork H. (2017). Laboratory investigation on unbound materials used in a highway with premature damage. Bearing capacity of Roads, Railways and Airfields.

[47] Arnevik A., Evensen R., Uthus N.S., Aurstad J., Aksnes J., Jørgensen T. (2018). Retningslinjer Asfalt 2019.

[48] Xu G., Wang H., Zhu H. (2017). Rheological properties and anti-aging performance of asphalt binder modified with wood lignin. Constr. Build. Mater..

[49] Yao H., You Z., Li L., Goh S.W., Lee C.H., Yap Y.K., Shi X. (2013). Rheological properties and chemical analysis of nanoclay and carbon microfiber modified asphalt with Fourier transform infrared spectroscopy. Constr. Build. Mater..

[50] Sabouri M., Mirzaiyan D., Moniri A. (2018). Effectiveness of Linear Amplitude Sweep (LAS) asphalt binder test in predicting asphalt mixtures fatigue performance. Constr. Build. Mater..

[51] Ameri M., Nowbakht S., Molayem M., Mirabimoghaddam M.H. (2016). A study on fatigue modeling of hot mix asphalt mixtures based on the viscoelastic continuum damage properties of asphalt binder. Constr. Build. Mater..

[52] Newcomb D. (2003). Limit the strain at the bottom of an asphalt pavement, and what do you get?: A perpetual pavement. HMAT Hot Mix. Asph. Technol..

[53] (2015). Bitumen and Bituminous Binders—Determination of the Softening Point—Ring and Ball Method.

[54] (2015). Bitumen and Bituminous Binders—Determination of Needle Penetration.

[55] Ongel A., Hugener M. (2015). Impact of rejuvenators on aging properties of bitumen. Constr. Build. Mater..

[56] Wang Z., Ye F. (2020). Experimental investigation on aging characteristics of asphalt based on rheological properties. Constr. Build. Mater..

[57] Mikhailenko P., Kou C., Baaj H., Poulikakos L., Cannone-Falchetto A., Besamusca J., Hofko B. (2019). Comparison of ESEM and physical properties of virgin and laboratory aged asphalt binders. Fuel.

[58] Boyko L.L. (1976). Microwave Heating for Road Maintenance.

[59] Scherocman J.A., Nath R.H. Hot Mix Recycling with Fluidized Bed and Microwave Heating. Proceedings of the 66th Annual Meeting of the Transportation Research Board.

[60] New Timehope WB Series Asphalt Road Microwave Maintenance Car. http://www.hnxth.com/english/product/prolist.jsp?myid=11246.

[61] World Health Organization Radiation: Microwave ovens. https://www.who.int/news-room/q-a-detail/radiation-microwave-ovens.

[62] Barbieri D.M., Lou B., Wang F., Hoff I., Wu S., Li J., Vignisdottir H.R., Bohne R.A., Anastasio S., Kristensen T. (2021). Assessment of carbon dioxide emissions during production, construction and use stages of asphalt pavements. Transp. Res. Interdiscip. Perspect..

[63] Butt A.A., Birgisson B., Kringos N. (2012). Optimizing the highway lifetime by improving the self healing capacity of asphalt. Procedia—Soc. Behav. Sci..

[64] Jeppson M. (1983). Microwave methods and apparatus for paving and paving maintenance. NASA STI/Recon. Tech. Rep. N.

[65] Association W.S. Statistical Reports. https://www.worldsteel.org/.

[66] Al-Mansoori T., Norambuena-Contreras J., Micaelo R., Garcia A. (2018). Self-healing of asphalt mastic by the action of polymeric capsules containing rejuvenators. Constr. Build. Mater..

[67] Su J.-F., Qiu J., Schlangen E. (2013). Stability investigation of self-healing microcapsules containing rejuvenator for bitumen. Polym. Degrad. Stab..

[68] Gallego J., del Val M.A., Contreras V., Paez A. (2017). Use of additives to improve the capacity of bituminous mixtures to be heated by means of microwaves. Mater. Constr..

[69] Lou B., Sha A., Barbieri D.M., Liu Z., Zhang F. (2021). Microwave heating properties of steel slag asphalt mixture using a coupled electromagnetic and heat transfer model. Constr. Build. Mater..

